# A joint analysis of influenza-associated hospitalizations and mortality in Hong Kong, 1998–2013

**DOI:** 10.1038/s41598-017-01021-x

**Published:** 2017-04-20

**Authors:** Peng Wu, Anne M. Presanis, Helen S. Bond, Eric H. Y. Lau, Vicky J. Fang, Benjamin J. Cowling

**Affiliations:** 1 WHO Collaborating Centre for Infectious Disease Epidemiology and Control, School of Public Health, Li Ka Shing Faculty of Medicine, The University of Hong Kong, Hong Kong Special Administrative Region, China; 2 Medical Research Council Biostatistics Unit, Cambridge Institute of Public Health, Cambridge, UK

## Abstract

Influenza viruses may cause severe human infections leading to hospitalization or death. Linear regression models were fitted to population-based data on hospitalizations and deaths. Surveillance data on influenza virus activity permitted inference on influenza-associated hospitalizations and deaths. The ratios of these estimates were used as a potential indicator of severity. Influenza was associated with 431 (95% CrI: 358–503) respiratory deaths and 12,700 (95% CrI: 11,700–13,700) respiratory hospitalizations per year. Majority of the excess deaths occurred in persons ≥65 y of age. The ratios of deaths to hospitalizations in adults ≥65 y were significantly higher for influenza A(H1N1) and A(H1N1)pdm09 compared to A(H3N2) and B. Substantial disease burden associated with influenza viruses were estimated in Hong Kong particularly among children and elderly in 1998–2013. Infections with influenza A(H1N1) was suggested to be more serious than A(H3N2) in older adults.

## Introduction

Influenza viruses circulate around the world each year, causing infections and disease in all age groups^1^. A small fraction of influenza virus infections are severe, requiring hospitalization, and some infections can be fatal. Apart from deaths caused by primary viral pneumonia, influenza virus infections can also lead to secondary bacterial infections^1^, and can exacerbate underlying medical conditions such as cardiovascular disease^2^. One approach to quantify the disease burden of influenza is to estimate the incidence rate of hospitalizations of patients with severe acute respiratory illnesses and laboratory-confirmed influenza virus infections, and the incidence of severe outcomes in those patients, along with population denominators^3^. However, this can underestimate the full burden of influenza because not all patients with severe outcomes after influenza virus infections present for medical attention or are tested for influenza virus, and because even among those that are tested some influenza virus infections will not be laboratory confirmed, particularly if there is a delay between infection and the exacerbation of an underlying condition^4^.

The preferred approach to quantify the full disease burden of influenza in a population is indirect estimation using statistical modeling^5^. In this ecological approach, a time series of hospitalization or mortality rates is regressed against a variable indicating influenza virus activity over time in the same population. This statistical model can then be used to infer the proportion of hospitalizations or deaths associated with influenza^6^. Statistical models can be fitted for time series of hospitalizations or deaths from specific causes, or all causes.

In the present study, we used hospitalization data, mortality data and influenza surveillance data from January 1998 through to June 2013 to infer the burden of influenza-associated hospitalizations and deaths in Hong Kong before, during and after the 2009–10 influenza A(H1N1)pdm09 pandemic. This updates our previous estimates of influenza-associated mortality from 1998–2009^7^ and in 2010–11^8^, as well as published estimates of influenza-associated hospitalizations in Hong Kong from 1996–2000^9^, 2004–10^10^, and 2005–10^11, 12^. In addition to estimating influenza-associated hospitalizations and deaths, we also examined the ratios of deaths to hospitalizations as a novel approach to indicate the potential relative severity of infections with different types/subtypes of influenza virus in a Bayesian framework.

## Methods

### Sources of data

Individual data on deaths were obtained from the Census and Statistics Department of Hong Kong. Each record included age, sex, date of death, and cause. Weekly data on hospital admissions were collected from the Hospital Authority for patients admitted to all local public hospitals, which cover approximately 90% of hospital bed days in Hong Kong^13^. Hospital admissions were aggregated by age, sex, date of admission, and primary diagnosis coded by the International Classification of Diseases, Ninth Revision, Clinical Modification (ICD-9-CM) including respiratory diseases (460–519), cardiovascular diseases (390–459), and all causes (001-999, V01-V91, E000-E999).

Influenza surveillance in local private outpatient clinics is coordinated by the Centre for Health Protection in Hong Kong, who report weekly data on the rates of influenza-like-illnesses (ILI, defined as fever >38 °C with cough and/or sore throat) per 1000 outpatient consultations. Age-specific ILI rates are not reported. Laboratory data on influenza virus detections by type/subtype and the total number of specimens submitted for diagnostic testing or for surveillance purposes are reported by the Public Health Laboratory Services. We combined outpatient surveillance data and laboratory detections into four time-series of proxy measures for influenza virus activity in the community, denoted as ILI+ proxies, by multiplying the rate of ILI consultations per 1000 consultations with the proportion of specimens testing positive for each type/subtype of influenza. We constructed ILI+ proxies for pre-pandemic influenza A(H1N1), influenza A(H3N2), influenza A(H1N1)pdm09, and for influenza B. We previously reported that this ILI+ proxy for influenza A(H1N1)pdm09 was the closest linear correlate of influenza A(H1N1)pdm09 virus infections in the general community^10^. We used the weekly hospitalization rate of acute bronchiolitis associated with respiratory syncytial virus (RSV) (ICD-9: 466.11) in children <1 year of age as the proxy for RSV activity^14^. Meteorological data including daily mean temperature and relative humidity were obtained from the Hong Kong Observatory.

### Statistical analysis

We applied linear regression models to investigate the association between influenza activity, as indicated by the ILI+ proxies, and weekly hospitalization rates or mortality rates. We used linear regression, assuming an additive relation between influenza activity and mortality rates or hospitalization rates, which might be preferred to models assuming a multiplicative increase in the outcomes associated with additive changes in influenza activity^6–8^. The models also included temperature, absolute humidity, and RSV activity using the RSV proxy described above. Another covariate was included into the models for mortality rates to account for the impact of the transition of coding system (ie, from ICD-9 to ICD-10) for mortality data, in Hong Kong since 2001^7^. We also included a covariate into the models for hospitalization rates to account for the impact of public holidays on hospital admissions. We excluded the time period when the outbreaks of severe acute respiratory syndrome (SARS) were reported from the analysis, i.e. from 11 February 2003 when the World Health Organization first received reports from China on an outbreak of acute respiratory syndrome occurred in Guangdong province, to 28 May 2004 when the Hong Kong government lowered the Alert Level of SARS response system.

The linear regression models (Supplementary Information) for estimation of influenza-associated excess mortality and hospitalization were fitted to the data in a Bayesian framework using Markov Chain Monte Carlo methods^15^. We chose to use the Bayesian framework for our analyses because this framework allows for propagation of uncertainty from the data through to all parameters and functions of parameters, including specifically the ratios of excess deaths to excess hospitalizations, and the comparison of these ratios between different influenza types and subtypes. It would be much more challenging to obtain uncertainty intervals for these ratios, and ratios-of-ratios, with an analysis done in a frequentist framework. Previous analyses of excess influenza-associated mortality have generally been done in frequentist frameworks, but one recent study did use a Bayesian framework^16^.

In each model, posterior distributions of the model parameters were estimated based on sampling from three Markov chains, each of which had 2000 iterations and a burn-in period of 500 iterations (Supplementary Information). Convergence was assessed using the potential scale reduction statistic^17^. Excess hospitalizations and deaths were estimated by comparing the predicted hospitalization and death rates under the model with ILI+ proxies set to zero with the predicted rates with ILI+ proxies set to their observed values^5, 7^. We estimated 95% credibility intervals for these estimates based on the samples drawn from the posterior distributions. Estimates of the ratios of deaths to hospitalizations, and comparisons of these ratios, were also based on the posterior samples. All statistical analyses were done in R version 3.2.3 (R Foundation for Statistical Computing, Vienna, Austria) and the ‘rstan’ package.

## Results

**Figure 1:**
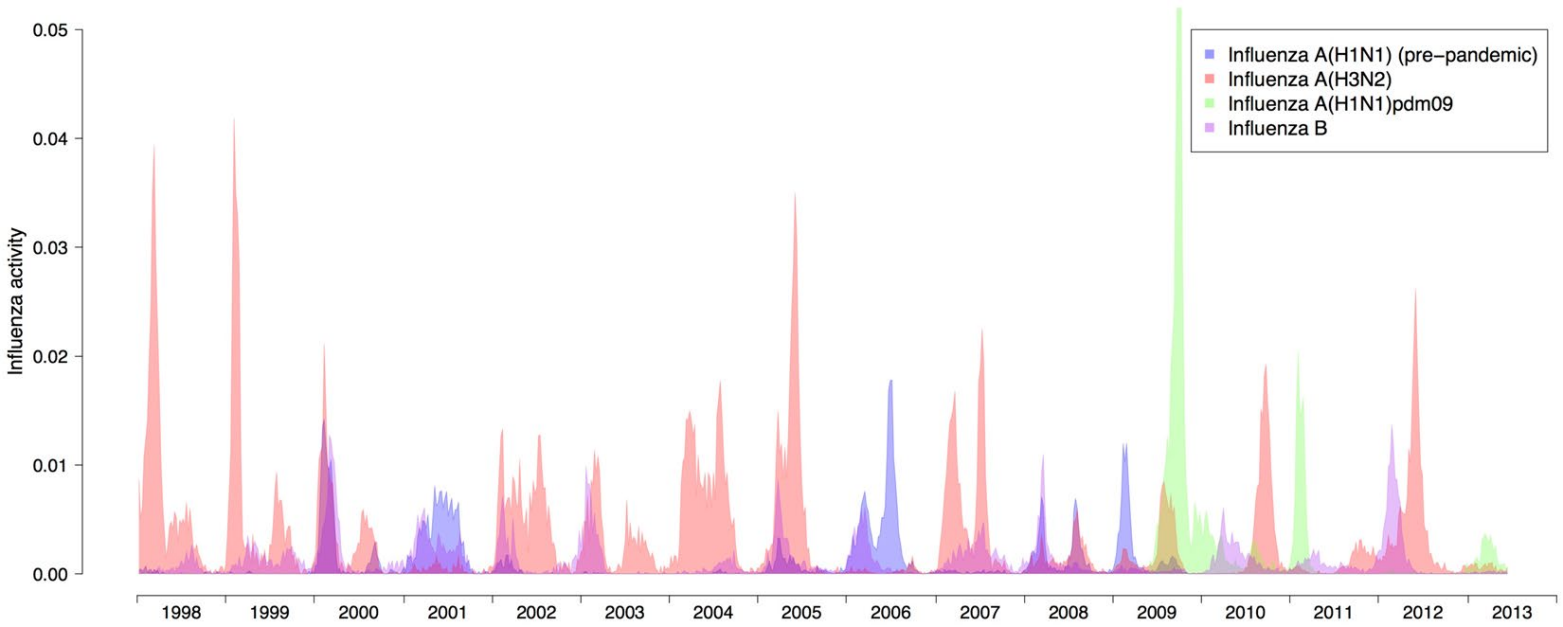
Weekly influenza virus activity in Hong Kong from 1998 through 2013, measured by ILI+ proxies defined for each influenza type/subtype as the product of the weekly proportion of outpatient consultations associated with influenza-like-illness in sentinel clinics and the weekly proportions of laboratory specimens testing positive for influenza A(H1N1), A(H3N2), A(H1N1)pdm09 and B viruses respectively.

We studied a total of 583,000 deaths and 18.9 million hospitalizations recorded from 1 January 1998 through 9 June 2013, with the population of Hong Kong increasing from 6.52 million persons in 1998 to 7.18 million in 2013. During the study period of 15.5 years, influenza viruses circulated for prolonged periods in most years, with two distinct epidemics in some years such as 2000, 2003 and 2008, and prolonged epidemics in other years such as 2002 and 2004 (Fig. 1).

Using regression analysis, we estimated that influenza was associated with 6.27 (95% credibility interval (CrI): 5.21, 7.32) excess respiratory deaths per 100,000 persons per year, and 184 (95% CrI: 170, 199) excess respiratory hospitalizations per 100,000 persons per year (Table 1). This corresponded to an average of 431 (95% CrI: 358, 503) excess respiratory deaths per year and an average of 12,700 (95% CrI: 11,700, 13,700) excess respiratory hospitalizations per year, which were 1.14% (85% CrI: 0.95%, 1.34%) of all deaths and 1.04% (95% CrI: 0.96%, 1.12%) of all hospitalizations over the study period, respectively.

**Table 1:** Average annual influenza-associated excess mortality and hospitalization rates from respiratory diseases by type and subtype of virus in different age groups of Hong Kong population from January 1998 through June 2013.

	<1 year	1–5 years	6–15 years	16–44 years	45–64 years	≥65 years		All ages
	Mean (95% credibility interval) (per 100,000 person-years)							
**Death**								
All influenza	2.2 (−0.2, 5.0)	−0.2 (−1.0, 0.5)	0.1 (−0.1, 0.4)	−0.1 (−0.2, 0.1)	1.8 (1.1, 2.5)	48.7 (40.4, 56.5)	6.3 (5.2, 7.3)	
A(H1N1	1.2 (−0.3, 2.8)	0.4 (−0.1, 0.8)	0.1 (−0.1, 0.2)	0.0 (−0.1, 0.1)	0.5 (0.0, 1.0)	9.2 (4.0, 14.1)	1.3 (0.7, 2.0)	
A(H3N2)	1.5 (0.0, 3.4)	−0.3 (−0.8, 0.2)	0.0 (−0.2, 0.2)	0.0 (−0.1, 0.1)	1.0 (0.5, 1.4)	26.5 (21.1, 31.9)	3.2 (2.6, 3.9)	
B	−0.1 (−1.9, 1.9)	−0.1 (−0.6, 0.5)	0.0 (−0.2, 0.2)	−0.1(−0.2, 0.1)	0.3 (−0.2, 0.9)	11.3 (5.0, 17.2)	1.4 (0.6, 2.3)	
A(H1N1)pdm09	−0.5 (−2.5, 1.4)	−0.5 (−1.0, 0.1)	0.1 (−0.1, 0.3)	0.1 (−0.1, 0.2)	0.4 (−0.1, 0.9)	13.1 (6.4, 19.4)	2.0 (1.2, 3.0)	
**Hospitalization**								
All influenza	1260.0 (1124.0, 1410.6)	692.0 (627.8, 760.3)	165.6 (148.4, 183.1)	15.5 (11.0, 19.9)	68.4 (60.6, 76.4)	847.2 (775.2, 913.2)	184.0 (167.0, 198.8)	
A(H1N1)	190.7 (112.4, 260.5)	87.0 (41.7, 127.7)	25.3 (16.6, 35.3)	2.7 (−0.3, 5.6)	7.6 (2.7, 12.4)	51.3 (7.6, 89.4)	17.3 (8.7, 26.4)	
A(H3N2)	648.7 (533.4, 738.0)	332.3 (288.3, 382.4)	49.1 (38.4, 60.6)	7.5 (4.2, 10.7)	36.2 (30.9, 41.0)	502.5 (454.0, 548.8)	97.9 (88.5, 106.4)	
B	379.6 (287.4, 468.7)	219.9 (161.1, 271.3)	62.9 (50.3, 76.9)	2.0 (−1.5, 5.7)	18.7 (12.8, 24.8)	276.0 (227.5, 325.4)	57.2 (45.0, 68.8)	
A(H1N1)pdm09	289.6 (187.8, 410.4)	249.5 (197.7, 301.1)	117.1 (103.5, 130.7)	13.3 (9.5, 16.9)	26.6 (20.7, 32.4)	99.8 (50.4, 150.6)	53.8 (42.0, 66.8)	

Rates of influenza-associated excess respiratory mortality were much greater in adults ≥65 y than in all other age groups, while rates of influenza-associated respiratory hospitalizations had a U-shaped relation with age, being greatest in those <1 y and ≥65 y, and lowest in those 16–44 y. Whereas 96% of excess respiratory deaths were estimated to occur in persons ≥65 y, only 57% of excess respiratory hospitalizations were estimated to occur in this age group.

When examining the contribution of different types and subtypes of influenza virus, we found that influenza A(H3N2) had the greatest impact, contributing around half of the excess respiratory mortality and respiratory hospitalizations on average, with influenza B contributing the second largest average during the study period of 15.5 years. Influenza A(H1N1)pdm09 was associated with the highest excess respiratory hospitalization rate in older children 6–15 y and young adults 16–44 y among all types/subtypes of influenza virus (Table 1).

We found that the proportions of cause-specific mortality and hospitalization attributable to influenza varied across different types/subtypes of influenza virus. The estimated contribution of influenza viruses to all-cause mortality and all-cause hospitalizations was approximately double the impact on respiratory mortality and hospitalizations respectively (Table 2). Influenza A(H3N2) was associated with approximately half of the influenza-attributable excess mortality and excess hospitalization across different causes of disease. Influenza B had a greater contribution to the excess all-cause hospitalizations (177 per 100,000 persons per year) than that to excess cardiorespiratory hospitalizations (75 per 100,000 persons per year), while cardiorespiratory diseases accounted for majority of excess hospitalizations for different subtypes of influenza A viruses (Table 2). Seasonal influenza A(H1N1) was generally associated with the lowest excess hospitalizations among the four types/subtypes of virus while the estimated excess mortality was largely similar to influenza B and A(H1N1)pdm09 across different causes of disease classifications.

**Table 2:** Average annual influenza-associated excess mortality and hospitalization rates from all causes, cardiovascular and respiratory diseases and pneumonia and influenza by virus type and subtype in Hong Kong population from January 1998 through June 2013.

	A(H1N1)	A(H3N2)	B	A(H1N1)pdm09	All influenza
	Mean (95% credibility interval) (per 100,000 person-years)				
**Death**					
All causes	2.3 (0.8, 3.8)	6.9 (5.4, 8.5)	2.4 (0.5, 4.5)	2.2 (−0.0, 4.2)	11.7 (9.2, 14.3)
Cardiovascular & respiratory	1.6 (0.6, 2.7)	4.9 (3.6, 6.0)	1.1 (−0.2, 2.4)	1.8 (0.4, 3.1)	7.7 (6.0, 9.6)
Respiratory	1.3 (0.7, 2.0)	3.2 (2.6, 3.9)	1.4 (0.6, 2.3)	2.0 (1.2, 3.0)	6.3 (5.2, 7.3)
Pneumonia and influenza	0.9 (0.4, 1.4)	1.8 (1.2, 2.3)	0.8 (0.1, 1.5)	1.5 (1.0, 2.2)	3.7 (2.8, 4.6)
**Hospitalization**					
All causes	−3.2 (−57.7, 44.4)	83.0 (24.9, 136.2)	177.2 (121.0, 234.3)	93.9 (36.4, 152.5)	284.9 (198.6, 357.8)
Cardiovascular & respiratory	19.5 (7.2, 31.0)	101.2 (89.5, 113.9)	75.2 (60.0, 90.7)	55.0 (37.9, 69.9)	207.5 (186.2, 227.8)
Respiratory	17.3 (8.7, 26.4)	97.9 (88.5, 106.4)	57.2 (45.0, 68.8)	53.8 (42.0, 66.8)	184.0 (170.0, 198.8)
Pneumonia and influenza	10.0 (5.6, 13.5)	38.4 (34.0, 43.3)	17.0 (11.8, 22.6)	38.2 (32.9, 43.6)	74.3 (67.6, 80.7)

We estimated that the ratio of excess respiratory deaths to excess respiratory hospitalizations was 34.2 (95% CrI: 27.6, 41.1) deaths per 1,000 hospitalizations in all ages for influenza viruses overall. In other words, for every 1,000 estimated excess respiratory hospitalizations associated with influenza, we estimated that there were around 34.2 influenza-associated excess respiratory deaths in Hong Kong. When stratifying analyses by age, the estimated ratios were unstable with wide credibility intervals in most age groups because of the low numbers of excess deaths, but we were able to estimate ratios for adults 45–64 y and ≥65 y. The ratios were smaller for 45–64 y, with an average of 26.5 (95% CrI: 15.6, 39.3) excess respiratory deaths for every one thousand excess respiratory hospitalizations in this age group, compared to 58.8 (95% CrI: 49.1, 69.1) excess respiratory deaths for every thousand excess respiratory hospitalizations in persons ≥65 y.

Estimates of excess death in persons ≥65 y allowed us to compare these ratios of deaths to hospitalizations between the different influenza types/subtypes. In this age group, the ratio of deaths to hospitalizations for H1N1pdm09 was a factor of 2.7 (95% CrI: 1.1, 5.9) higher than the ratio of deaths to hospitalzations for H3N2 (Table 3). Similarly, the hospitalization to death ratio was significantly higher for seasonal H1N1 compared to H3N2 by a factor of 3.6 (95% CrI: 1.4, 14.5), and was significantly higher for H1N1pdm09 compared to influenza B by a factor of 3.8 (95% CrI: 1.2, 10.0), and for seasonal H1N1 compared to influenza B by a factor of 5.8 (95% CrI: 1.4, 25.8). There was no significant difference in the death to hospitalization ratio for influenza B compared to H3N2 (ratio 0.8, 95% CrI: 0.4, 1.4) and for H1N1pdm09 compared to seasonal H1N1 (ratio 1.8, 95% CrI: 0.4, 7.5). We were unable to evaluate similar ratios for other age groups because of the greater uncertainty in the estimated death-to-hospitalization ratios.

**Table 3:** Ratios of the influenza-associated annual excess respiratory death rate to the excess respiratory hospitalization rate by virus type/subtype and relative ratios of excess respiratory death to hospitalization for influenza A(H1N1), A(H1N1pdm09) and B compared to influenza A(H3N2) in Hong Kong population ≥65 years of age from January 1998 through June 2013.

	Ratios of excess respiratory death to hospitalization	Relative ratios of excess respiratory death to hospitalization
	Mean (95% credibility interval) (per 1,000 excess hospitalizations)	Mean (95% credibility interval)
All influenza	58.8 (49.1, 69.1)	—
Influenza by type/subtype		
A(H3N2)	52.5 (40.6, 65.3)	Reference
A(H1N1)	193.8 (74.2, 754.5)	3.6 (1.4, 14.5)
A(H1N1)pdm09	141.4 (57.6, 294.5)	2.7 (1.1, 5.9)
B	41.7 (19.3, 68.2)	0.8 (0.4, 1.4)

## Discussion

In our study, we estimated that a substantial burden of morbidity and mortality in Hong Kong population could be attributed to influenza virus infections during our study period of 1998–2013, including around 1.0% of all hospitalizations and 1.1% of all deaths. The two extremes of age, 0–5 years and ≥65 years, were most vulnerable to severe respiratory influenza virus infections. However the highest rates of excess respiratory mortality occurred in older adults ≥65 years accounting for around 1.4% of all-cause deaths in that age group, while influenza-associated excess mortality in children was close to zero, as was also shown in a recent study conducted in the United Kingdom^18^. The higher impact of influenza on mortality among the elderly might be associated with a higher prevalence of underlying medical conditions in this segment of the population^19, 20^. The estimates of the highest excess respiratory hospitalization in young children were largely consistent with findings from other studies of influenza-associated hospital admissions in Hong Kong^21, 22^.

Influenza A(H3N2) is generally found to cause the greatest impact among all influenza types/subtypes, because of its particularly high impact in older adults^5, 7^. The impact of each type/subtype will vary from year to year because of different patterns in circulation, but our study over 15.5 years covered a broad timespan with more than one influenza epidemic in most years (Fig. 1). Our results indicated that influenza A(H3N2) consistently contributed around half of the overall excess hospitalizations across different age groups except for among children 6–15 y in which most influenza-associated excess hospitalizations were due to H1N1pdm09 during the study period. The relatively higher estimates of excess hospital admission associated with A(H1N1)pdm09 compared to seasonal A(H1N1) perhaps implied a higher disease burden introduced by A(H1N1)pdm09 since the seasonal influenza A(H1N1) virus was replaced by A(H1N1)pdm09 after 2009.

In persons ≥65 y, we estimated that for every 1000 influenza-associated excess hospitalizations there were 58.8 (95% CrI: 49.1, 69.1) deaths. Because not every death would have necessarily been preceded by a hospitalization, we cannot interpret this estimate as a risk of death among older adults hospitalized with influenza. Nevertheless, the estimate is well within the range of 50 to 100 deaths per 1000 hospitalizations that would have been anticipated based on analyses of cohorts of hospitalized older adults in which the risk of mortality is typically around 4–8%^23–25^.

We compared the estimates of excess respiratory death to excess respiratory hospitalization ratios for persons ≥65 y between influenza types and subtypes, and found that the ratios were significantly higher for pre-pandemic influenza A(H1N1) and A(H1N1)pdm09 viruses, compared to influenza A(H3N2) and B (Table 3). While A(H1N1) viruses tended to have lower overall impact in older adults (Table 1), our analysis on excess death to hospitalization ratios suggests the possibility that influenza A(H1N1) virus infections are relatively more severe, on a per-infection basis, than influenza A(H3N2) and B virus infections. This hypothesis is supported by analyses of hospitalized patients in Hong Kong and the United States, where the risk of complications was higher for influenza A(H1N1)pdm09 virus infections compared to seasonal influenza virus infections, after adjusting for age^23, 26^.

An advantage of conducting these analyses within a Bayesian framework was the availability of uncertainty ranges for ratios of estimates, such as death to hospitalization ratios. In a frequentist framework, it can be challenging to assess the uncertainty of estimates based on combinations of other parameters. The Bayesian framework for this analysis might also be extended to allow timely estimation of disease impact in very recent years before the availability of hospitalization and mortality data, by using most recent data to update the posterior distributions of parameter estimates from prior years.

There are a number of limitations to our work. First, hospital admission data collected from all the public hospitals in Hong Kong were used to estimate influenza-associated excess respiratory hospitalizations, which might lead to an underestimation of excess respiratory hospitalizations because 90% of hospital admissions occurred in public hospitals in Hong Kong while data on the other 10% occurring in private hospitals are unavailable. Second, we estimated that there were very few excess respiratory deaths associated with influenza in children and younger adults, while excess hospitalization rates were low in older children and younger adults, precluding estimation of death to hospitalization ratios for these age groups. Third, in this study we only estimated an averaged influenza-associated impact across age groups although other risk factors such as underlying medical conditions, pregnancy or previous vaccination history might potentially modify the impact from influenza on the population. Finally, we examined excess respiratory deaths and respiratory hospitalizations, which may underestimate the full burden of influenza because of its impact on other diseases such as cardiovascular diseases^27, 28^ or other diseases^5, 7^. Previous studies have often estimated that the impact of influenza virus epidemics on all-cause mortality is around double or triple the impact on respiratory mortality^5, 29, 30^.

In conclusion, influenza viruses caused substantial excess respiratory hospitalizations particularly in children and the elderly in Hong Kong in 1998–2013, while most influenza-associated excess respiratory deaths occurred in persons ≥65 y. Seasonal influenza A(H3N2) accounted for majority of the excess disease burden, while our estimates of death to hospitalization ratios suggested that infections with seasonal influenza A(H1N1) and A(H1N1)pdm09 viruses might be more serious on average compared to other virus types/subtypes, in persons ≥65 y. Further studies would be warranted to investigate the differential impact of influenza on particular segments of the population that might face a higher risk of severe disease following infection, such as pregnant women, and adults with underlying medical conditions including COPD or chronic heart diseases.

## Electronic supplementary material

SUPPLEMENTARY INFORMATION
