# Supplementary material for: A joint analysis of influenza-associated hospitalizations and mortality in Hong Kong, 1998–2013
Source: Sci Rep. 2017 Apr 20;7:929. doi: 10.1038/s41598-017-01021-x (PMC5430505; doi:10.1038/s41598-017-01021-x)
Supplement: Supplementary file 1 — SUPPLEMENTARY INFORMATION [file 41598_2017_1021_MOESM1_ESM.doc]

**SUPPLEMENTARY INFORMATION**

**A joint analysis of influenza-associated hospitalizations and mortality in Hong Kong, 1998-2013**

Peng Wu, Anne M. Presanis, Helen S. Bond, Eric H. Y. Lau, Vicky J. Fang, Benjamin J. Cowling

This supplementary information provides additional technical details of the statistical methods used in this study.

1. **Linear regression model**

Generalized additive models were applied to examine the association between influenza virus activity and the outcomes of interest including all-cause and respiratory hospitalization rates and death rates accounting for other potential confounding factors. The following two equations show the models used for weekly death and hospitalization rates.

*μ_D_ = β_1_*s(Week Index, k=30) + β_2_* (h1n1*ILI) + β_3_* (h3n2*ILI) + β_4_* (B*ILI) + β_5_* (ph1n1*ILI) + β_6_* (ph1n1*ILI*pandemic) + β_7_* (RSV*ILI) + β_8_*TEMP + β_9_*TEMP^2^ + β_10_*TEMP^3^ + β_11_* AH + β_12_*AH^2^ + β_13_*AH^3^ + β_14_*NameChange*

*Death rate ~ Normal(μ_D_, τ_D_^2^)*

*μ_H_ = α_1_*s(Week Index, k=30) + α_2_* (h1n1*ILI) + α_3_* (h3n2*ILI) + α_4_* (B*ILI) + α_5_* (ph1n1*ILI) + α_6_* (ph1n1*ILI*pandemic) + α_7_* (RSV*ILI) + α_8_*TEMP + α_9_*TEMP^2^ + α_10_* TEMP^3^ + α_11_*AH + α_12_*AH^2^ + α_13_*AH^3^ + α_14_*Holiday*

*Hospitalization rate ~ Normal(μ_H_, τ_H_^2^)*

Where,

- *s(Week Index, k=30)* is a cubic regression spline on the calendar weeks of the study period with 31 knots (2 per year).
- *pandemic* is a dummy variable accounting for potential changes in public health responses to the 2009 pandemic during the first wave of the pandemic, as used in previous studies ^1,2^ with the same proxy for influenza activity that would otherwise overestimate the influenza H1N1pdm09 activity during the peak of the first wave compared to other periods.
- *TEMP* is the mean weekly temperature.
- *AH* is the mean weekly absolute humidity.
- *NameChange* is a dummy variable accounting for the change in disease coding system for mortality data from ICD-9 (1998-2000) to ICD-10 (2001 and thereafter) in Hong Kong (0 before change, 1 after change). In exploratory analysis it was noted that there were step changes in some cause-specific mortality time series on this date that were likely to be due to the change in coding and categorization of some deaths.
- *Holiday* is a dummy variable in the model for hospitalizations to allow for possible differences in health seeking behavior of the public during public holidays (0 for non-holiday days, 1 for public holiday days).
- *β_1_ - β_14_* are the regression coefficients for variables included in the mortality model.
- *α_1_ - α_14_* are the regression coefficients for variables included in the hospitalization model.

1. **Bayesian framework of the analysis**

Bayesian inference is a framework for statistical inference in which we can update any prior information with observed data to obtain posterior estimates of parameter values. Bayes theorem, which underlies Bayesian inference, can be expressed as:
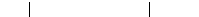
, where
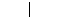
 is the posterior distribution of the parameters of interest,
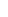
, given the data, which is proportional to the likelihood function for the observed data,
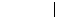
 multiplied by the prior information on parameter values
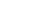
.

We chose to use the Bayesian framework for our analyses because it allows for propagation of uncertainty from the data through to all parameters and functions of parameters, including specifically the ratios of excess deaths to excess hospitalizations and the comparison of these ratios between different influenza types and subtypes. It would be much more complex to obtain uncertainty intervals for these ratios and ratios-of-ratios with an analysis done in a frequentist framework.

The priors specified in our Bayesian model were the following:

***β_i_*** *~ normal(0,10), i = 1,… , 14*, independently for each regression coefficient in the death model, chosen as weakly-informative priors ^3^ to stabilize the model.

***α_i_*** *~ normal(0,10) , i = 1,… , 14*, independently for each regression coefficient in the hospitalization model, chosen as weakly-informative priors ^3^ to stabilize the model.

*τ_D_ ~ Cauchy(0,5)*, where *τ_D_* is the scale parameter for the death model, chosen as a half-Cauchy prior to give a weakly-informative prior restricting the parameter to positive values ^3^. The half-Cauchy prior distribution was chosen based on recommendations that this is preferable to a uniform prior (which can over-represent the likelihood of large variances and cause stability issues), and preferable to a gamma distribution which can be too informative ^3,4^.

*τ_H_ ~ Cauchy(0,5)*, where *τ_H_* is the scale parameter for the hospitalization model, chosen as a half-Cauchy prior to give a weakly-informative prior restricting the parameter to positive values ^3^.

Once the priors were chosen, posterior distributions of the model parameters were estimated using a Markov chain Monte Carlo simulation approach.

1. **Modeling using *Stan* in R**

The program used to conduct the Bayesian analysis was the *Stan* package in R. *Stan* provides full Bayesian inference through Markov Chain Monte Carlo (MCMC) methods, using an adapted form of Hamiltonian Monte Carlo sampling (the No-U-turn sampler**.** MCMC algorithms generate random samples from the joint posterior distribution of interest, which are summarized by their means and 95% credible intervals. Hamiltonian Monte Carlo is an efficient MCMC algorithm that uses geometric methods to accelerate convergence to the posterior distribution and subsequent sampling from the posterior.

**References**

1 Wu, P. *et al.* Excess mortality associated with influenza A and B virus in Hong Kong, 1998-2009. *J Infect Dis* **206**, 1862-1871, doi:10.1093/infdis/jis628 (2012).

2 Tsang, T. K. *et al.* Interpreting seroepidemiologic studies of influenza in a context of nonbracketing sera. *Epidemiology* **27**, 152-158, doi:10.1097/EDE.0000000000000408 (2016).

3 Gelman, A. Prior distributions for variance parameters in hierarchical models (comment on article by Browne and Draper). *Bayesian Analysis* **1**, 515-534, doi:10.1214/06-BA117A (2006).

4 Polson, N. G. & Scott, J. G. On the half-Cauchy prior for a global scale parameter. *Bayesian Analysis* **7**, 887-902, doi:10.1214/12-BA730 (2012).

Figure S1. Weekly all-cause (top) and respiratory (bottom) death rates recorded by the death registry (grey dots), in comparison to the predicted weekly all-cause (top) and respiratory (bottom) death rates from the regression models with (black) and without (blue) influenza activity in Hong Kong population from 1998 through 2013.

Figure S2. Weekly all-cause (top) and respiratory (bottom) hospitalization rate recorded by the Hospital Authority (grey dots), in comparison to the predicted weekly all-cause (top) and respiratory (bottom) hospitalization rates from the regression models with (black) and without (blue) influenza activity in Hong Kong population from 1998 through 2013.

Table S1. Estimated coefficients for the variables included in the regression model for estimation the influenza-associated respiratory mortality rates (model equation see Supplementary Information) in Hong Kong, 1998-2013.

|  | **Regression coefficient (95% CrI)** | | | | | |
| --- | --- | --- | --- | --- | --- | --- |
|  | **<1 year** | **1-5 years** | **6-15 years** | **16-44 years** | **45-64 years** | **≥65 years** |
| Intercept | 160  (150, 170) | 85  (73, 91) | 18  (16, 20) | 4.5  (4, 5.2) | 14  (12, 17) | 150  (140, 150) |
| Spline beta1_1_ | 10  (-7.2, 28) | 11  (-4.2, 30) | 0.58  (-1.2, 1.8) | -0.087  (-1.3, 1.1) | 2.6  (-0.13, 5.6) | -24  (-36, -12) |
| Spline beta1_2_ | 37  (24, 52) | 11  (-2.3, 22) | -0.49  (-2.2, 0.69) | 0.17  (-0.61, 0.99) | 3.7  (1.4, 6.2) | 2.3  (-5, 9.3) |
| Spline beta1_3_ | -6  (-10, 6.8) | -3.9  (-13, 3.9) | -1.3  (-3.2, 0.64) | 0.25  (-0.5, 0.96) | 4.2  (1.8, 6.8) | 9.4  (1.7, 17) |
| Spline beta1_4_ | 18  (4.6, 31) | 2.6  (-3.3, 11) | -0.16  (-1.8, 0.62) | -0.22  (-1.2, 0.39) | 3.1  (0.87, 5.8) | 0.33  (-8.8, 7.9) |
| Spline beta1_5_ | -0.27  (-13, 13) | -1.4  (-12, 7.4) | -0.86  (-3. 1.6) | 0.12  (-0.59, 1) | 3.6  (1.2, 6.2) | 13  (4.7, 21) |
| Spline beta1_6_ | 15  (2, 27) | 2.7  (-1.9, 12) | -0.85  (-3, 1.4) | 0.19  (-0.79, 0.98) | 4.5  (2.1, 7.1) | 21  (14, 28) |
| Spline beta1_7_ | 13  (0.61, 26) | 2.7  (-4.9, 12) | 0.3  (-2.7, 3.7) | 0.23  (-0.5, 1.1) | 2.9  (0.65, 5.5) | 11  (3, 18) |
| Spline beta1_8_ | 34  (21, 47) | 9.5  (-1.3, 19) | 0.6  (-1.6, 3.4) | 0.35  (-1.3, 1.6) | 2.9  (0.5, 5.4) | 26  (18, 33) |
| Spline beta1_9_ | -1.8  (-14, 12) | 0.47  (-4.6, 6.6) | -1.6  (-3.4, 0.15) | -0.0025  (-0.6, 0.58) | 1.8  (-0.49, 4.3) | 24  (16, 32) |
| Spline beta1_10_ | 7.1  (-5.6, 20) | 4.4  (-2, 12) | -1.5  (-3.9, 0.062) | -0.29  (-1.5, 0.53) | 2  (-0.41, 4.6) | 24  (17, 32) |
| Spline beta1_11_ | -22  (-35, -8.3) | -8.3  (-18, 1.2) | -1.1  (-3.6, 3.3) | -0.39  (-1.4, 0.34) | -0.9  (-3.3, 1.7) | -1.5  (-9.1, 6.8) |
| Spline beta1_12_ | -29  (-42, -16) | -18  (-31, -0.57) | -1.2  (-5.1, 1.8) | -1.2  (-2.2, -0.51) | -2.5  (-4.8, 0.18) | -15  (-23, -7) |
| Spline beta1_13_ | -20  (-33, -6.8) | -18  (-26, -0.43) | -2.5  (-6.9, 3) | -0.36  (-1.3, 1.3) | -1.4  (-3.7, 0.73) | 1.9  (-5.4, 9.2) |
| Spline beta1_14_ | 3  (-11, 16) | -8.1  (-17, 1.1) | -0.33  (-2.2, 1.2) | -0.87  (-1.9, 0.27) | -2.1  (-4.5, 0.39) | 9.1  (1.6, 16) |
| Spline beta1_15_ | -8.5  (-22, 5.8) | -2.3  (-8.5, 4.2) | -0.7  (-2.5, 1.6) | -0.92  (-1.6, -0.13) | -3.1  (-5.4, -0.56) | -11  (-18, -3.4) |
| Spline beta1_16_ | 17  (3.3, 32) | -0.15  (-6.5, 6.6) | 0.46  (-2, 2.3) | -0.9  (-1.8, 0.052) | -3.7  (-6.1, -1.2) | -16  (-24, -8.5) |
| Spline beta1_17_ | -0.89  (-14, 12) | -4.8  (-13, 1.2) | -1.5  (-2.5, -0.72) | -0.65  (-1.4, 0.052) | -2.4  (-4.8, 0.12) | 2.4  (-4.1, 9.6) |
| Spline beta1_18_ | 21  (7.8, 33) | -4.7  (-13, 3.9) | -1.3  (-6.1, 2.8) | -0.6  (-1.7, 0.86) | -3.4  (-5.7, -0.89) | -10  (-18, -3.5) |
| Spline beta1_19_ | 3.2  (-9.9, 16) | -1.8  (-10, 5) | -0.32  (-2.8, 1.8) | -0.91  (-1.5, -0.29) | -2.8  (-5.1, -0.5) | 2.8  (-3.8, 9.5) |
| Spline beta1_20_ | 16  (3.4, 29) | 2.4  (-3.5, 7.5) | -0.18  (-1.5, 2) | -0.66  (-1.3, 0.17) | -2.1  (-4.4, 0.43) | 8.9  (1.2, 16) |
| Spline beta1_21_ | 11  (-2.4, 24) | -0.55  (-5.1, 6.1) | -1  (-2.3, 0.6) | -0.9  (-1.7, -0.24) | -2.8  (-5.2, -0.31) | 3.9  (-2.5, 12) |
| Spline beta1_22_ | -13  (-26, 0.86) | -13  (-21, 0.77) | 0.83  (-0.65, 3.5) | 1.1  (0.2, 1.7) | -0.97  (-3.3, 1.5) | 2.3  (-5, 9.9) |
| Spline beta1_23_ | 11  (-2.5, 25) | 11  (1.4, 24) | 0.8  (-0.34, 3.2) | 0.62  (-0.062, 1.5) | 0.6  (-1.8, 3.2) | 12  (3.1, 21) |
| Spline beta1_24_ | -2.1  (-16, 12) | 9  (1.4, 19) | 0.99  (-1.4, 3.6) | 0.67  (-0.5, 1.4) | 0.17  (-2.2, 2.7) | 20  (12, 28) |
| Spline beta1_25_ | 2.7  (-12, 17) | 11  (-0.71, 19) | 0.81  (-2.8, 5.1) | 0.6  (-0.2, 1.1) | -1.6  (-4, 0.98) | 5.3  (-2.7, 13) |
| Spline beta1_26_ | 12  (-0.33, 25) | 4.1  (-2.2, 14) | 0.76  (-2.2, 3.1) | 0.76  (-0.35, 2) | -0.62  (-2.9, 1.9) | 13  (5, 20) |
| Spline beta1_27_ | -4.3  (-17, 11) | 5.5  (-2.5, 15) | 1.5  (-1.3, 6.1) | 0.17  (-0.94, 1.1) | -1.1  (-3.4, 1.4) | -9.1  (-16, -1.3) |
| Spline beta1_28_ | 5.8  (-7, 19) | 12  (-1.1, 24) | 3.7  (-1.1, 9) | 0.91  (-0.13, 1.9) | 0.53  (-1.9, 3.1) | 18  (11, 25) |
| Spline beta1_29_ | 19  (5.3, 32) | 9.7  (0.24, 19) | 0.72  (-0.12, 2.2) | 0.21  (-0.56, 0.83) | -0.76  (-3.1, 1.9) | -6.8  (-15, -0.31) |
| Spline beta1_30_ | 12  (-5.3, 29) | 15  (-0.065, 35) | 3.7  (0.58, 9.5) | 1.4  (1, 2) | 1.5  (-1.1, 4.2) | 7.3  (-3, 17) |
| Beta2 | 31  (18, 43) | 12  (2.4, 21) | 2.8  (1.3, 5.2) | 0.48  (-0.15, 1.4) | 1.2  (0.52, 1.9) | 8.8  (2.4, 15) |
| Beta3 | 33  (28, 39) | 16  (12, 21) | 1.8  (1.1, 2.6) | 0.43  (0.23, 0.62) | 1.9  (1.7, 2.2) | 27  (24, 29) |
| Beta4 | 27  (16, 40) | 18  (2.1, 27) | 2.3  (-0.94, 5) | 1.2  (0.21, 2.4) | 5.7  (4.9, 6.6) | 21  (13, 29) |
| Beta5 | -13  (-25, -1.2) | -4.3  (-16, 6.9) | 5.1  (0.62, 8) | -0.87  (-2.2, 0.18) | -5.6  (-6.5, -4.7) | -21  (-29, -13) |
| Beta6 | 57  (44, 71) | 31  (9.5, 41) | 1.9  (-0.85, 5.8) | 0.69  (0.13, 1.2) | 3.4  (2.7, 4.2) | 44  (36, 52) |
| Beta7 | 87  (70, 100) | 15  (3.4, 26) | 0.85  (-0.89, 1.7) | 0.89  (0.052, 1.7) | 1.9  (0.2, 3.5) | 33  (21, 46) |
| Beta8 | 0.24  (-2.9, 3.3) | 2.9  (0.79, 4.6) | -0.89  (-1.6, -0.39) | -0.0086  (-0.068, 0.056) | -0.12  (-0.23, -0.0015) | -4.3  (-5.6, -3.1) |
| Beta9 | -0.17  (-0.46, 0.12) | -0.26  (-0.42, 0.033) | -0.016  (-0.064, 0.057) | -0.0031  (-0.01, 0.0037) | -0.013  (-0.025, 0.00072) | 0.066  (-0.053, 0.19) |
| Beta10 | 0.034  (-0.00043, 0.07) | -0.0071  (-0.024, 0.019) | 0.005  (0.000016, 0.012) | 0.00017  (-0.00048, 0.0008) | 0.0019  (0.00047, 0.0032) | 0.036  (0.022, 0.05) |
| Beta11 | -0.012  (-3.2, 3) | -3  (-4.8, -1.1) | 0.4  (-0.34, 1) | 0.03  (-0.03, 0.09) | -0.0028  (-0.11, 0.12) | 1.1  (-0.18, 2.3) |
| Beta12 | -0.078  (-0.076, 0.0031) | -0.054  (-0.19, 0.15) | 0.067  (0.031, 0.14) | 0.0013  (-0.0047, 0.0074) | 0.0077  (-0.004, 0.019) | 0.049  (-0.06, 0.16) |
| Beta13 | -0.037  (-0.076, 0.0031) | 0.0013  (-0.018, 0.035) | 0.0055  (-0.0014, 0.016) | -0.00065  (-0.0014, 0.000042) | -0.00096  (-0.0025, 0.00045) | -0.022  (-0.038, -0.0073) |
| Beta14 | 23  (17, 30) | 2.3  (-1.1, 9) | -0.13  (-1.2, 1.9) | -0.19  (-0.36, -0.07) | -0.36  (-0.62, -0.094) | 1.9  (-0.85, 4.7) |

Note: The data was transformed before running the STAN model to speed up running time – running time is faster if all data is of the same order of magnitude. Therefore the following transformations were made:

- ILI and all flu subtypes multiplied by 100.
- Temperature, humidity and week index centered.

Table S2. Estimated coefficients for the variables included in the regression model for estimation the influenza-associated respiratory hospitalization rates (model equation see Supplementary Information) in Hong Kong, 1998-2013.

|  | **Regression coefficient (95% CrI)** | | | | | |
| --- | --- | --- | --- | --- | --- | --- |
|  | **<1 year** | **1-5 years** | **6-15 years** | **16-44 years** | **45-64 years** | **≥65 years** |
| Intercept | -0.72  (-4.1, 2.8) | -0.23  (-1.7, 1.5) | 0.1  (-0.19, 0.36) | 0.0044  (-0.16, 0.25) | 0.57  (-0.073, 1.3) | 11  (7.1, 15) |
| Spline alpha1_1_ | 0.76  (-2.9, 4.2) | 0.35  (-1.4, 1.9) | -0.09  (-0.35, 0.2) | 0.029  (-0.22, 0.19) | 0.0023  (-0.76, 0.65) | -0.54  (-4.7, 3.4) |
| Spline alpha1_2_ | 0.9  (-2.6, 4.3) | 0.29  (-1.4, 1.8) | -0.068  (-0.32, 0.22) | 0.064  (-0.18, 0.23) | 0.17  (-0.61, 0.83) | 3.8  (-0.36, 7.9) |
| Spline alpha1_3_ | 0.82  (-2.7, 4.3) | 0.27  (-1.5, 1.8) | -0.075  (-0.33, 0.21) | 0.043  (-0.2, 0.2) | 0.047  (-0.74, 0.69) | 3  (-1.1, 7) |
| Spline alpha1_4_ | 0.98  (-2.6, 4.5) | 0.29  (-1.4, 1.8) | -0.075  (-0.34, 0.21) | 0.03  (-0.21, 0.19) | -0.042  (-0.82, 0.62) | 0.79  (-3.4, 4.7) |
| Spline alpha1_5_ | 0.68  (-2.9, 4.1) | 0.26  (-1.5, 1.8) | -0.092  (-0.34, 0.2) | 0.047  (-0.2, 0.21) | -0.092  (-0.89, 0.56) | -0.72  (-4.8, 3.1) |
| Spline alpha1_6_ | 0.75  (-2.8, 4.2) | 0.24  (-1.5, 1.8) | -0.079  (-0.34, 0.21) | 0.038  (-0.2, 0.2) | -0.038  (-0.82, 0.61) | 1.7  (-2.3, 5.6) |
| Spline alpha1_7_ | 0.75  (-2.8, 4.2) | 0.24  (-1.5, 1.8) | -0.068  (-0.31, 0.21) | 0.025  (-0.22, 0.18) | -0.29  (-1, 0.3) | -3.1  (-6.6, 0.43) |
| Spline alpha1_8_ | 0.85  (-2.8, 4.2) | 0.05  (-1.7, 1.6) | -0.045  (-0.29, 0.24) | 0.018  (-0.23, 0.17) | -0.28  (-1, 0.34) | -0.78  (-4.2, 2.6) |
| Spline alpha1_9_ | 0.76  (-2.5, 4.4) | 0.11  (-1.6, 1.6) | -0.044  (-0.29, 0.24) | 0.0075  (-0.23, 0.16) | -0.33  (-1.1, 0.28) | -1.8  (-5.4, 1.7) |
| Spline alpha1_10_ | 0.62  (-2.7, 4.2) | 0.098  (-1.7, 1.6) | -0.055  (-0.3, 0.23) | 0.024  (-0.22, 0.17) | -0.36  (-1.1, 0.27) | -1.6  (-5.2, 2.1) |
| Spline alpha1_11_ | 0.86  (-2.8, 4.2) | 0.15  (-1.6, 1.7) | -0.059  (-0.31, 0.23) | 0.005  (-0.24, 0.16) | -0.37  (-1.1, 0.23) | -2.4  (-6.1, 1) |
| Spline alpha1_12_ | 0.61  (-2.5, 4.4) | 0.09  (-1.7, 1.6) | -0.057  (-0.3, 0.22) | 0.027  (-0.21, 0.18) | -0.34  (-1.1, 0.25) | -0.98  (-4.5, 2.5) |
| Spline alpha1_13_ | 0.7  (-2.8, 4.2) | 0.21  (-1.5, 1.7) | -0.046  (-0.29, 0.24) | 0.016  (-0.22, 0.17) | -0.35  (-1.1, 0.25) | -1.4  (-4.9, 2.1) |
| Spline alpha1_14_ | 0.68  (-2.7, 4.2) | 0.1  (-1.7, 1.6) | -0.047  (-0.29, 0.24) | 0.031  (-0.21, 0.18) | -0.39  (-1.2, 0.22) | 1.1  (-2.7, 4.6) |
| Spline alpha1_15_ | 0.72  (-2.8, 4.2) | 0.13  (-1.6, 1.6) | -0.033  (-0.28, 0.26) | 0.021  (-0.22, 0.17) | -0.36  (-1.1, 0.24) | -1.3  (-5, 2.1) |
| Spline alpha1_16_ | 0.74  (-2.7, 4.2) | 0.08  (-1.7, 1.6) | -0.053  (-0.3, 0.23) | 0.017  (-0.23, 0.17) | -0.45  (-1.2, 0.18) | -0.65  (-4.2, 2.8) |
| Spline alpha1_17_ | 0.59  (-2.8, 4.2) | 0.093  (-1.7, 1.6) | -0.052  (-0.31, 0.23) | 0.024  (-0.22, 0.17) | -0.31  (-1.1, 0.3) | -0.17  (-3.8, 3.3) |
| Spline alpha1_18_ | 0.62  (-2.9, 4) | 0.1  (-1.6, 1.6) | -0.071  (-0.32, 0.21) | 0.014  (-0.22, 0.16) | -0.4  (-1.2, 0.2) | 1  (-2.6, 4.5) |
| Spline alpha1_19_ | 0.74  (-2.9, 4.1) | 0.14  (-1.6, 1.7) | -0.057  (-0.31, 0.23) | 0.028  (-0.22, 0.18) | -0.36  (-1.1, 0.23) | 0.68  (-3.2, 4.1) |
| Spline alpha1_20_ | 0.82  (-2.8, 4.2) | 0.15  (-1.6, 1.6) | -0.055  (-0.3, 0.23) | 0.023  (-0.22, 0.17) | -0.37  (-1.1, 0.25) | 2.5  (-1.2, 6.2) |
| Spline alpha1_21_ | 0.72  (-2.7, 4.3) | 0.084  (-1.7, 1.6) | -0.051  (-0.29, 0.23) | 0.018  (-0.22, 0.17) | -0.37  (-1.1, 0.23) | 0.78  (-2.9, 4.4) |
| Spline alpha1_22_ | 0.73  (-2.7, 4.2) | 0.062  (-1.7, 1.6) | -0.059  (-0.31, 0.23) | 0.019  (-0.22, 0.17) | -0.42  (-1.2, 0.19) | 0.79  (-2.8, 4.4) |
| Spline alpha1_23_ | 0.76  (-2.7, 4.2) | 0.16  (-1.6, 1.7) | -0.042  (-0.29, 0.24) | 0.046  (-0.2, 0.2) | -0.27  (-1, 0.33) | 2.2  (-1.5, 6) |
| Spline alpha1_24_ | 1  (-2.6, 4.3) | 0.14  (-1.6, 1.6) | -0.07  (-0.32, 0.21) | 0.018  (-0.22, 0.17) | -0.31  (-1.1, 0.3) | 2.1  (-1.6, 5.5) |
| Spline alpha1_25_ | 0.71  (-2.5, 4.6) | 0.16  (-1.6, 1.6) | -0.057  (-0.31, 0.23) | 0.024  (-0.22, 0.18) | -0.32  (-1.1, 0.29) | 0.84  (-2.8, 4.4) |
| Spline alpha1_26_ | 0.69  (-2.7, 4.1) | 0.16  (-1.6, 1.7) | -0.062  (-0.32, 0.22) | 0.021  (-0.22, 0.17) | -0.32  (-1.1, 0.29) | 2.7  (-0.89, 6.1) |
| Spline alpha1_27_ | 0.98  (-2.7, 4.1) | 0.12  (-1.6, 1.6) | -0.051  (-0.3, 0.24) | 0.042  (-0.2, 0.19) | -0.33  (-1.1, 0.28) | 0.075  (-3.9, 3.7) |
| Spline alpha1_28_ | 0.54  (-2.5, 4.5) | 0.13  (-1.6, 1.6) | -0.059  (-0.3, 0.22) | 0.021  (-0.22, 0.17) | -0.34  (-1.1, 0.27) | 4.3  (0.72, 7.7) |
| Spline alpha1_29_ | 0.79  (-2.9, 4) | 0.13  (-1.6, 1.6) | -0.038  (-0.29, 0.25) | 0.016  (-0.23, 0.17) | -0.32  (-1.1, 0.28) | 0.98  (-2.8, 4.5) |
| Spline alpha1_30_ | 0.71  (-2.7, 4.3) | 0.045  (-1.7, 1.6) | -0.028  (-0.27, 0.26) | 0.025  (-0.22, 0.17) | -0.31  (-1.1, 0.31) | 3.1  (-0.7, 6.9) |
| Alpha2 | 0.22  (-2.8, 4.3) | 0.065  (-0.015, 0.14) | 0.013  (-0.01, 0.036) | -0.0011  (-0.019, 0.017) | 0.065  (-0.0043, 0.13) | 1.3  (0.56, 2.1) |
| Alpha3 | 0.085  (-0.0058, 0.18) | -0.0097  (-0.034, 0.016) | -0.00024  (-0.0084, 0.0081) | -0.00013  (-0.0064, 0.0059) | 0.054  (0.027, 0.078) | 1.4  (1.1, 1.7) |
| Alpha4 | -0.036  (-0.37, 0.28) | -0.076  (-0.18, 0.026) | 0.029  (-0.00053, 0.06) | 0.022  (-0.0011, 0.045) | 0.11  (0.025, 0.2) | 2.7  (1.7, 3.7) |
| Alpha5 | -0.0036  (-0.35, 0.35) | 0.063  (-0.05, 0.17) | -0.023  (-0.058, 0.0077) | -0.025  (-0.05, 0.00039) | -0.12  (-0.21, -0.027) | -2.6  (-3.6, -1.5) |
| Alpha6 | -0.099  (-0.42, 0.22) | -0.024  (-0.11, 0.061) | 0.0081  (-0.02, 0.035) | 0.00015  (-0.019, 0.018) | 0.084  (-0.0042, 0.17) | 1.9  (0.92, 2.8) |
| Alpha7 | 0.034  (-0.55, 0.65) | 0.051  (-0.12, 0.22) | -0.02  (-0.078, 0.037) | 0.02  (-0.02, 0.058) | -0.024  (-0.2, 0.14) | 1.1  (-0.67, 2.8) |
| Alpha8 | -0.023  (-0.07, 0.023) | -0.0073  (-0.02, 0.0058) | -0.00019  (-0.0042, 0.0041) | -0.0012  (-0.0043, 0.0017) | -0.012  (-0.024, 0.0013) | -0.41  (-0.56, -0.28) |
| Alpha9 | 0.0059  (0.0013, 0.01) | -0.00067  (-0.0019, 0.00069) | 0.00005  (-0.00035, 0.00044) | 0.00008  (-0.00021, 0.0004) | 0.00044  (-0.00088, 0.0017) | 0.022  (0.0085, 0.036) |
| Alpha10 | 0.00054  (0.000028, 0.0012) | 0.000072  (-0.000085, 0.00022) | -0.00003  (-0.000077, 0.000018) | 0.000011  (-0.000025, 0.000046) | 0.000077  (-0.00008, 0.00022) | 0.0012  (-0.00041, 0.0028) |
| Alpha11 | 0.0017  (-0.044, 0.05) | 0.006  (-0.0073, 0.02) | 0.001  (-0.003, 0.005) | 0.00051  (-0.0024, 0.0037) | 0.003  (-0.01, 0.015) | 0.047  (-0.095, 0.21) |
| Alpha12 | -0.0027  (-0.0067, 0.0012) | 0.00093  (-0.00022, 0.0022) | -0.00023  (-0.00063, 0.00012) | -0.000096  (-0.00037, 0.00016) | 0.0004  (-0.00076, 0.0016) | 0.0013  (-0.011, 0.013) |
| Alpha13 | -0.000062  (-0.00065, 0.00055) | -0.00011  (-0.00029, 0.000073) | -0.000011  (-0.000064, 0.000045) | -0.0000097  (-0.00045, 0.00027) | -0.000024  (-0.00019, 0.00014) | -0.0022  (-0.0039, -0.00045) |
| Alpha14 | 0.046  (-0.67, 0.67) | 0.17  (-0.023, 0.35) | -0.032  (-0.09, 0.028) | 0.016  (-0.024, 0.058) | 0.13  (-0.059, 0.3) | 2.3  (0.45, 4.3) |

Note: The data was transformed before running the STAN model to speed up running time – running time is faster if all data is of the same order of magnitude. Therefore the following transformations were made:

- ILI and all flu subtypes multiplied by 100.

- Temperature, humidity and week index centered.
